# GelMA hydrogel dual photo-crosslinking to dynamically modulate ECM stiffness

**DOI:** 10.3389/fbioe.2024.1363525

**Published:** 2024-06-20

**Authors:** Josephina J. H. M. Smits, Atze van der Pol, Marie José Goumans, Carlijn V. C. Bouten, Ignasi Jorba

**Affiliations:** ^1^ Department of Biomedical Engineering, Eindhoven University of Technology, Eindhoven, Netherlands; ^2^ Institute for Complex Molecular Systems (ICMS), Eindhoven, Netherlands; ^3^ Department of Cell and Chemical Biology, Leiden University Medical Centre, Leiden, Netherlands; ^4^ Unitat de Biofísica i Bioenginyeria, Facultat de Medicina i Ciències de la Salut, Universitat de Barcelona, Barcelona, Spain

**Keywords:** GelMA, mechanical memory, cardiac fibroblast (cFb), ECM, stiffness, tissue engineeering, photo-crosslinking

## Abstract

The dynamic nature of the extracellular matrix (ECM), particularly its stiffness, plays a pivotal role in cellular behavior, especially after myocardial infarction (MI), where cardiac fibroblasts (cFbs) are key in ECM remodeling. This study explores the effects of dynamic stiffness changes on cFb activation and ECM production, addressing a gap in understanding the dynamics of ECM stiffness and their impact on cellular behavior. Utilizing gelatin methacrylate (GelMA) hydrogels, we developed a model to dynamically alter the stiffness of cFb environment through a two-step photocrosslinking process. By inducing a quiescent state in cFbs with a TGF-β inhibitor, we ensured the direct observation of cFbs-responses to the engineered mechanical environment. Our findings demonstrate that the mechanical history of substrates significantly influences cFb activation and ECM-related gene expression. Cells that were initially cultured for 24 h on the soft substrate remained more quiescent when the hydrogel was stiffened compared to cells cultured directly to a stiff static substrate. This underscores the importance of past mechanical history in cellular behavior. The present study offers new insights into the role of ECM stiffness changes in regulating cellular behavior, with significant implications for understanding tissue remodeling processes, such as in post-MI scenarios.

## Introduction

The extracellular matrix (ECM) is inherently dynamic, with its properties, including stiffness, undergoing significant variations in response to both physiological and pathological stimuli (Lu et al., 2011; Hu et al., 2022). For instance, after myocardial infarction (MI) there is considerable ECM remodeling of the cardiac tissue, with associated changes in ECM composition and structure. This remodeling is mainly orchestrated by the activation and proliferation of cardiac fibroblasts (cFbs) (van den Borne et al., 2010). cFbs aid in replacing the damaged tissue with abundant ECM leading to a fibrotic scar, resulting in a stiffer environment compared to the healthy one.

Nowadays it is well acknowledged that cells actively respond to the mechanical properties of their surrounding tissue (Obbink-Huizer et al., 2014; Holle et al., 2018; Zhang et al., 2020; Jorba et al., 2021; Mostert et al., 2022; Jorba et al., 2024). As such, ECM stiffness is a potent cue to activate cFbs towards a profibrotic, high-ECM producing phenotype (Gałdyszyńska et al., 2021). Therefore, ECM stiffness is not just a result of tissue remodeling but also a contributing factor to the formation of fibrotic tissue post-MI. *In vitro* studies show that cFbs cultured on top of stiff substrates show a high expression of stress fibers directly associated with a higher cFb activation and ECM producing cell. In contrast, cFbs cultured on top of soft substrates exhibit a quiescent, low-producing ECM phenotype, typically found in healthy myocardium (Verma et al., 2023; Felisbino et al., 2024). Emerging research has shown that cells not only respond to their immediate mechanical environment but also retain a ‘memory’ of past mechanical signals. This mechanical memory can influence subsequent cellular responses such as proliferation, migration, and differentiation, even when the current mechanical cues differ from past experiences (Yang et al., 2014; Nasrollahi et al., 2017; Dudaryeva et al., 2023; Scott et al., 2023). Despite its importance in cellular behavior, the effect of dynamic stiffness changes on cFb phenotype is not well understood.

Typically, *in vitro* studies to study cellular mechanical memory consist of culturing cells on substrates of specific (static) stiffness and then transferring them to substrates of different stiffness to observe changes in behavior (Yang et al., 2014; Dunham et al., 2020). However, these approaches cannot capture the inherent ECM stiffness dynamics mimicking *in vivo* conditions. More recently, significant efforts have been made to develop stimulus-responsive biomaterials. These dynamic materials are usually composed of ECM molecules and stimuli-responsive motifs, whereas stimuli such as temperature, light, magnetic force, or biomolecules have been employed to trigger continuous changes in biomaterial properties (Xie et al., 2023). Photoresponsive biomaterials have emerged as promising platforms to dynamically alter stiffness in 2D and 3D set-ups due to their remote manipulability and the high spatial and temporal control over stiffness changes (Lee et al., 2018). In particular, ECM natural proteins such as hyaluronic acid or gelatin have been modified using photoresponsive methacrylate groups (Bencherif et al., 2008; Yue et al., 2015). UV-light exposure at any desired time results in hydrogel crosslinking and thus an increase in substrate stiffness (Yue et al., 2015; Chalard et al., 2022). For instance, hyaluronic acid methacrylate (HAMA) temporal photo-crosslinking has been used to study the effects of dynamic ECM stiffness on cancer cell migration and phenotype (Ondeck et al., 2019). The much-used gelatin methacrylate (GelMA) hydrogels, however, have not been reported in studying the dynamics of ECM stiffness by temporal photo-crosslinking.

In this study, we use temporal GelMA photocrosslinking to dynamically change the stiffness of the cFbs environment. This approach allows us to investigate how previous ECM stiffness levels affect cFbs activation and ECM production at the gene level, filling a gap in our understanding of ECM stiffness dynamics and their impact on cellular behavior.

## Methods

### Experiment overview

GelMA hydrogels, dynamically stiffened, underwent two UV illumination steps for photocrosslinking: initially at 0 h and subsequently at 24 h. These gels were prepared in Polydimethylsiloxane (PDMS) molds to ensure uniform shapes ideal for cellular culture. For the cell experiments, cFbs were seeded at 0 h, following the first illumination, across all conditions. They were then exposed to the second illumination at different time points depending on the group type. Experimental groups and performed experiments are detailed in Supplementary Table S1.

### Fabrication of the casting molds

PDMS molds were created according to the procedure listed by SYLGARD™ 184 Silicone Elastomer Kit. The created PDMS was poured into a 2 mm or 5 mm (for cell seeding experiments) even layer before being placed in a vacuum to remove any air bubbles and followed by solidifying in a 60°C oven. Hollow cylindrical PDMS molds were created by cutting out a disk using an 8 mm diameter bio punch (Kai Medical), followed by a smaller 5 mm diameter bio punch (Kai Medical) to create the inner cavity. These molds were stored in 0.1% w/v Pluronic F-127 (Sigma Aldrich) in MilliQ water at RT overnight before use.

### Preparation of GelMA hydrogels

GelMA (Sigma-Aldrich) was dissolved in PBS 1x at w/v ratios of 5%, 10%, and 15%. To photocrosslink, a 2% lithium phenyl-2,4,6 trimethyl-benzoylphosphinate (LAP; Sigma Aldrich) in PBS 1x stock solution was created, afterwards stored at 4°C in the dark, and used in a final concentration in each hydrogel of 0.1%. 35 μL of the GelMA-LAP pregel were casted into the PDMS molds to provide for a cylindrical gel for cFbs culture. In all conditions, independent of the mold height, hydrogels were cast with a height of 2 mm. Samples were illuminated by UV-light of 365 nm wavelength (Analytik Jena UVP XX-15L; 5 mW/cm^2^). For samples where later illumination was required to dynamically change substrate stiffness, culture medium supplemented with a 0.1% w/v LAP was added to the samples to incubate for 45 min in the dark, before starting UV-light exposure.

### Stiffness measurements

To determine the stiffness of GelMA hydrogels, the hydrogels were measured by Micro-Indentation (CellScale) using a spherical indenter probe (radius: 0.5 mm). Force-indentation (F-δ) curves were recorded at 37°C PBS bath. F-δ curves were measured at 0.02 mm/s (and tip total displacement of 0.4 mm) and until a maximum indentation of 0.2 mm (∼10% of sample height). Five repeated measurements were recorded. The mean of the loading curves was taken to calculate the Young’s modulus (E), using the Hertzian equation (Eq. 1) (Jorba et al., 2017).

F=4E31−ν2R12δ32 (1)

### Sol fraction and swelling ratio

To determine the sol fraction and swelling ratio, samples were prepared as described before. Samples were then freeze-dried (Zirbus Vaco 2 freeze dryer; ZIRBUS Technology GmbH) overnight and weighted (W_i_). Hereafter, samples were kept overnight at 37°C in PBS and weighed (W_s_). Immediately after, the samples were again freeze-dried overnight and weighted (W_d_). The sol fraction (SF) and swelling ratio (q) were then determined by Eqs 2 and 3:

$$SF\;\left(\%\right)=\frac{W_{i}-W_{d}}{W_{s}}*100$$ (2)

$$q=\frac{W_{s}-W_{d}}{W_{d}}$$ (3)

### Scanning electron microscopy (SEM)

To assess the potential changes in hydrogel surface, SEM images were performed after hydrogel crosslinking. The hydrogels were fixed in 4% paraformaldehyde (PFA) in PBS overnight and then washed three times with PBS. Next, the samples were incubated in 4% osmium tetroxide for 90 min and then rinsed with deionized water. Subsequently, samples were dehydrated by washing them with ethanol 80% (×2), 90% (×3), 96% (×3), and 100% (×3) and preserved in absolute ethanol at 4°C until critical point drying (Autosamdri-815 critical point dryer, Tousimis, Rockville, MD, USA). Samples were then carbon-coated and mounted using conductive adhesive tabs (TED PELLA, Redding, CA, USA). Imaging was performed by using an SEM (JSM-6510, JEOL, Tokyo, Japan) at 15 kV.

### Human cFb culture

Human cFbs were derived from foetal epicardium. Human foetal cardiac tissue was anonymously collected with informed consent from elective abortion material of foetuses. Foetal epicardial layers were isolated by separating the epicardium from the underlying myocardium of human hearts aged 14–19 weeks post-gestation (Dronkers et al., 2018). This research was carried out according to the official guidelines of the Leiden University Medical Center and approved by the local Medical Ethics Committee (No. P08.087). cFbs were cultured with high-glucose Dulbecco’s modified Eagle’s medium (DMEM, Invitrogen), supplemented with 10% fetal bovine serum (FBS, SERANA) and 1% Pen/Strep (Gibco). The cFbs were cultured in T75 and/or T25 flasks coated with 0.1% gelatin from porcine skin (Sigma Aldrich) in PBS. Passaging of the cFbs took place after obtainment of a minimum of 80% confluency with the use of 0.05% Tripsin-EDTA (Gibco). cFbs cultured on top of the GelMA hydrogels were plated at a density of 500 cells/mm^2^, except for RT-qPCR that was 3,000 cells/mm^2^.

### cFb quiescence induction

Two approaches were used to induce quiescence in cFbs: 1) Reducing FBS content in the cFbs growth medium and 2) TGF-β inhibitor (SB431542 Sigma-Aldrich). The first approach consisted in reducing the FBS content in the growth medium from the standard 10% to 2% and 0%. Therefore, cFbs were cultured in DMEM culture medium supplemented with variable FBS concentration, 1% Pen/Strep. The second approach was an adapted version of an established protocol as described by Zhang et al. (Zhang et al., 2019). Briefly, cFbs were cultured on plastic for 7/8 days in DMEM culture medium supplemented with 2% FBS, 1% Pen/Strep and varying concentrations of TGF-β inhibitor of 0, 10 and 20 μM. Afterwards, cell expression of α-SMA were checked with a fluorescence microscope (Leica DMi8). When using the generated quiescent cFb on GelMA hydrogels, cells were cultured in DMEM supplemented only with 2% FBS and 1% Pen/Strep.

### Immunostaining

Samples of cells on plastic were washed with PBS 1x before being fixed in 3.7% para-formaldehyde (PFA) in PBS for 5 min. After fixation, samples were rewashed with PBS, before being stored in PBS at 4°C. Samples of cells on different GelMA stiffness groups were washed with PBS before being fixed in 3.7% formaldehyde for PFA in PBS for 30 min. All samples were incubated with primary antibodies (αSMA, A2547 Sigma) overnight at 4°C in the dark and with secondary antibody (Alexa 488, A21131 Molecular Probes) for 1 h at room temperature in the dark. Any non-antibody dyes were added to the secondary antibody staining mix. Phalloidin 550 (Sigma 19,083, 0.18 nM) stained the cFbs actin cytoskeleton. A DAPI (Sigma D9542, 0.39 mg/mL) staining was used to visualize the nuclei of the cFbs. Images were taken with a fluorescence microscope (Leica DMi8) using either a ×5 objective, ×10 objective or a ×20 objective.

### cFb viability

To assess the viability of cFbs after UV-light exposure, cFbs cultures on GelMA hydrogels were carefully washed with PBS and incubated with 1 μg/mL calcein AM and 750 nM propidium iodide (Invitrogen) for 20 min at 37°C protected from light. After incubation, cFb cultures were washed with PBS and directly imaged with an inverted microscope (Leica DMi8, Leica, Mannheim, Germany) using the ×10 objective.

### Western blotting

Protein pellet lysates were collected by treating cells with RIPA lysis buffer (Thermo Fisher), after which the cells were scraped off the well-bottom and homogenization via pipetting took place. Subsequently, protein measurement was performed according to the BCA Protein Assay (Pierce). Afterwards, denaturation of the proteins took place by heating the samples for 10 min at 95°C. Of each sample, 5 μg was loaded onto a 10% SDS-Page gel, together with Precision plus protein dual color standard (Biorad) ladder and run at 120 V for 1.5 h. Samples were transferred to a 0.45 μm nitrocellulose (GE Healthcare Life Science) blotting membrane, after which the membranes were blocked in 5% milk in PBS-Tween (0.1%) and then incubated with primary antibody (αSMA, A2547 Sigma; β-tubulin, E7 DSHB) overnight at 4°C and with secondary antibody (HRP, 31,457 Pierce) for 1 h at room temperature. Chemiluminescent Reagent Supersignal West Fempto (Thermo Scientific) was applied to the membrane for 2 min, after which the membrane was illuminated on the iBright 1,500 (Thermo Fisher).

### RT-qPCR

Cells were lysed using TRIZOL (Thermo Fischer) and either scraped off the well plate or crushed with their gel. Afterwards, lysates were homogenized by repeatedly pipetting up and down. Subsequently, RNA purification was performed using chloroform, isopropanol and various centrifuging steps. Purified RNA was dissolved in RNAse-free water, and concentrations were measured using Nanodrop one (Thermo Scientific). Reverse transcription was performed with Quantitect RT kit (Qiagen) as the manufacturer’s protocol instructed. Samples with 1,000 nM primers (Supplementary Table S2) and iQ SYBR Green Supermix (Biorad) were analyzed with RT-qPCR (CFX 384 Touch Real-Time PCR Detection System; Bio-Rad). Gene expression was determined by correcting for reference gene values (GAPDH), and the values were calculated by means of the delta-delta Ct method.

### Statistical analysis

Data are presented as the mean ± SEM (n = 3–5) was performed. Analyses were performed with GraphPad Prism 10.1.0 software (GraphPad Prism), and *p* < 0.05 was considered significant.

## Results

### GelMA mechanical characterization

**FIGURE 1:**
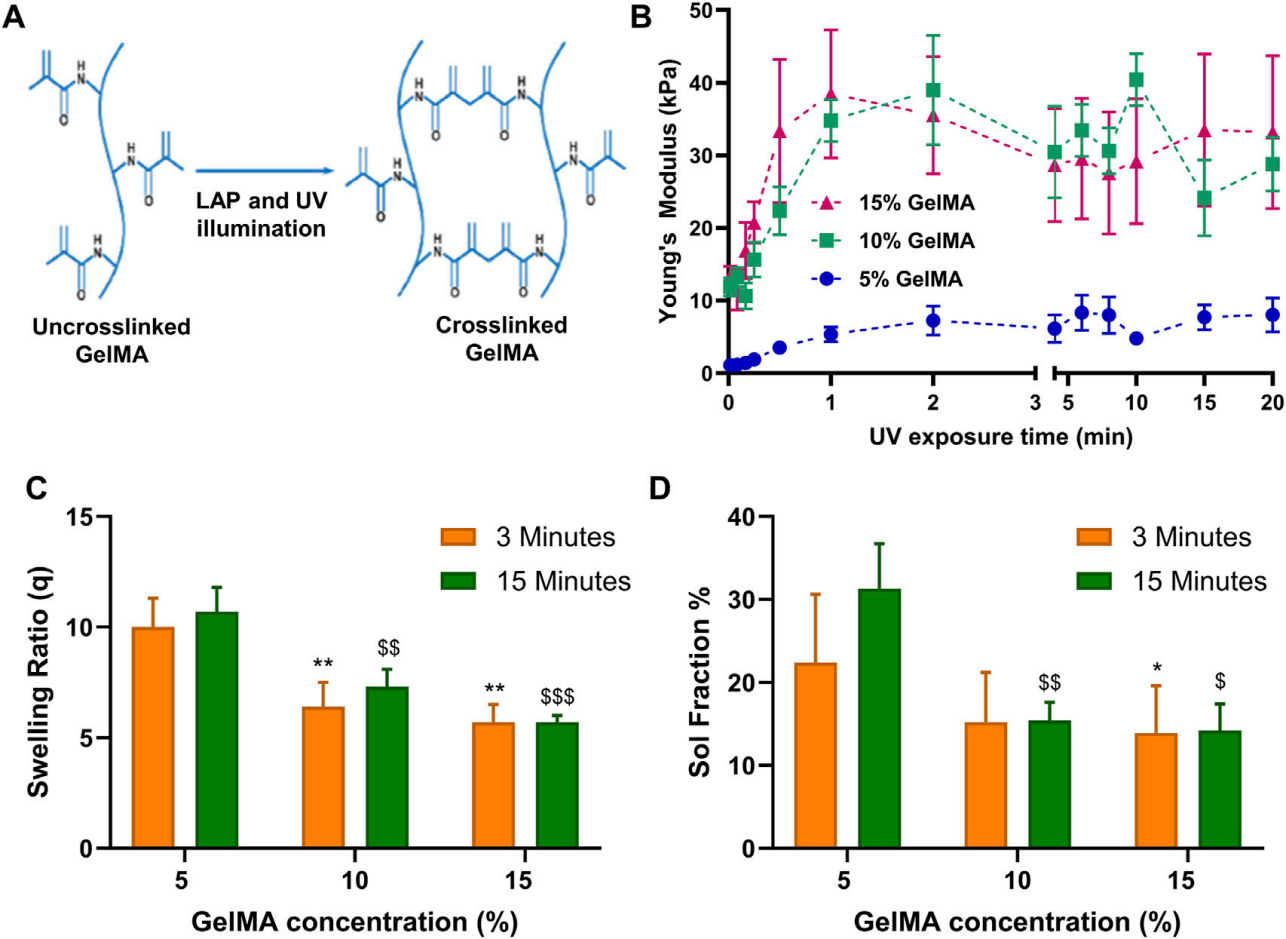
Characterization of the effects of GelMA crosslinking. **(A)** Schematics of the GelMA reaction crosslinking. **(B)** GelMA stiffness as function of UV exposure time and GelMA concentration (n = 5). **(C)** Swelling ratio of GelMA 10% gels exposed 3 and 15 min. **(D)** Sol fraction (%) of GelMA 10% gels exposed 3 and 15 min. One-way ANOVA was used for **(C, D)**. *, $ indicates significant difference compared to 5% GelMA concentration for 3 min and 15 min UV illumination, respectively.

GelMA stiffness tunability is achieved through the formation of covalent bonds between methacryolyl groups attached to the gelatin backbone, a process activated by a photoinitiator under UV light exposure (Figure 1A). Here, three GelMA concentrations (5%, 10%, and 15% w/v in PBS 1x) were examined to determine the relationship between GelMA concentration, UV illumination time, and resultant stiffness (Figure 1B). Our findings revealed that 5% GelMA hydrogels exhibited significantly lower stiffness, with a maximum of approximately 8 kPa, compared to the 10% and 15% concentrations, which both reached stiffness levels around 35 kPa. Notably, no discernible stiffness difference was observed between the 10% and 15% GelMA gels across varying illumination times, which is similar to what another study found (Chalard et al., 2022). Moreover, all GelMA concentrations achieved maximum stiffness within a time frame of 2–5 min of UV exposure. The stiffer gels (10% and 15% GelMA) had lower swelling ratios and sol fractions (i.e., the proportion of unreacted methacryolyl groups available for crosslinking), in comparison to the 5% GelMA hydrogels (Figures 1C, D). Our data specifically highlight that the 10% GelMA concentration stands out as the most effective, offering balance between stiffness tunability and polymer concentration.

### GelMA hydrogels can be stiffened over time

**FIGURE 2:**
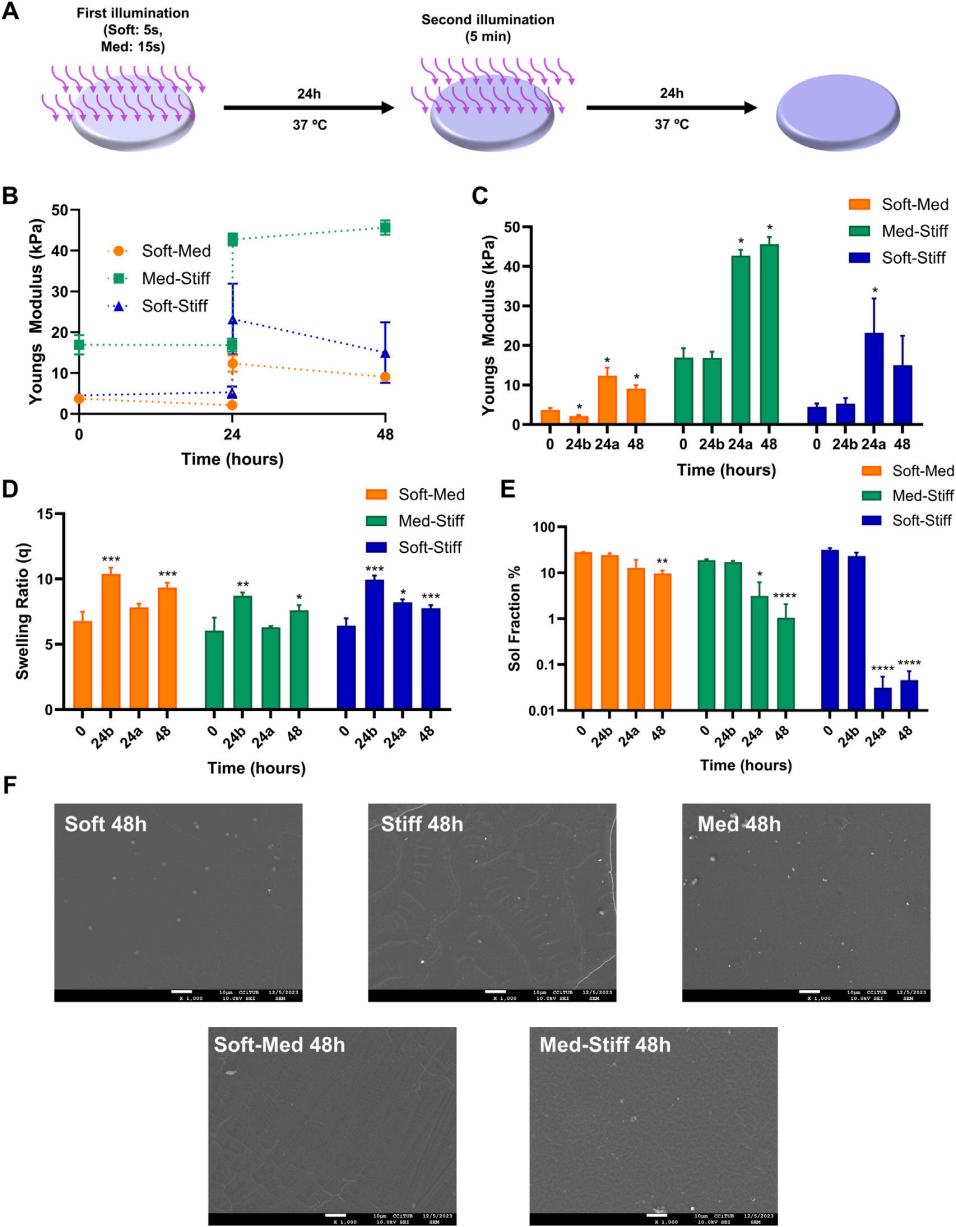
Two-step photocroslinking of GelMA hydrogels. **(A)** Followed protocol to dynamically stiffen GelMA hydrogels. **(B)** Stiffness of hydrogels at different timepoints. **(C)** Bar graph representation of the data at **(B)**. **(D)** Swelling ratio of GelMA hydrogels. **(E)** Sol fraction. **(F)** SEM images of GelMA hydrogel surface (n = 1). n = 4 for stiffness, swelling and sol fraction experiments. One-way ANOVA was used in C, D and E for each of the stiffness groups. *, **, ***, **** indicates significance compared to the 0 h group.

Taking into account that GelMA 10% concentration was the most suitable to achieve a wider range of stiffnesses, we used GelMA 10% for the subsequent experiments. One of the objectives of the study was to demonstrate that GelMA hydrogels can be stiffened over time. For that, the followed protocol was to initially illuminate the hydrogel at 0 h at leave it in cell culture medium for 24 h at 37°C. Thereafter, more LAP was added 45 min prior to the second illumination. The gels were kept for another 24 h inside the incubator (Figure 2A). Control hydrogels were only illuminated at 0 h and then kept at 37°C for 48 h. GelMA hydrogel stiffness was measured just after the first illumination (0 h), before the second illumination (24 h b), after the second illumination (24 h a), and 24 h after the second illumination (48 h) (Figure 2B). Tuning the first illumination time from 5 s to 15 s allowed to generate soft and medium stiffness (soft and med) groups that had a stiffness of ∼5 kPa and ∼18 kPa, respectively. The second illumination of 5 min allowed to stiffen the soft hydrogels reaching a stiffness of ∼12 kPa (Soft-Med) and ∼22 kPa (Soft-Stiff) followed by a slight decrease of both groups at 48 h to a stiffness of ∼18 kPa. Additionally, the second illumination of the Med hydrogel of 5 min allowed to stiffen the hydrogel up to ∼40 kPa (Med-Stiff) and stabilizing the stiffness at 48 h at ∼43 kPa (Figure 2C). Swelling ratio of the hydrogels were found to correlate with their crosslinking degree, displaying a reduction in swelling as sol fraction decreased and stiffness increased (Figures 2D,E). Given the similarity in stiffness results between the Soft-Med and Soft-Stiff groups, the Soft-Stiff group was excluded from further experiments. Lastly, SEM imaging was performed to evaluate any topographical changes in the hydrogel surfaces. These images did not show qualitative differences between hydrogels (Figure 2F), suggesting that stiffness alterations do not cause structural changes on the hydrogel surface. In summary, these results demonstrate the capability of GelMA hydrogels to be effectively stiffened over time through a two-step photocrosslinking procedure.

### Induction of cFbs quiescence

**FIGURE 3:**
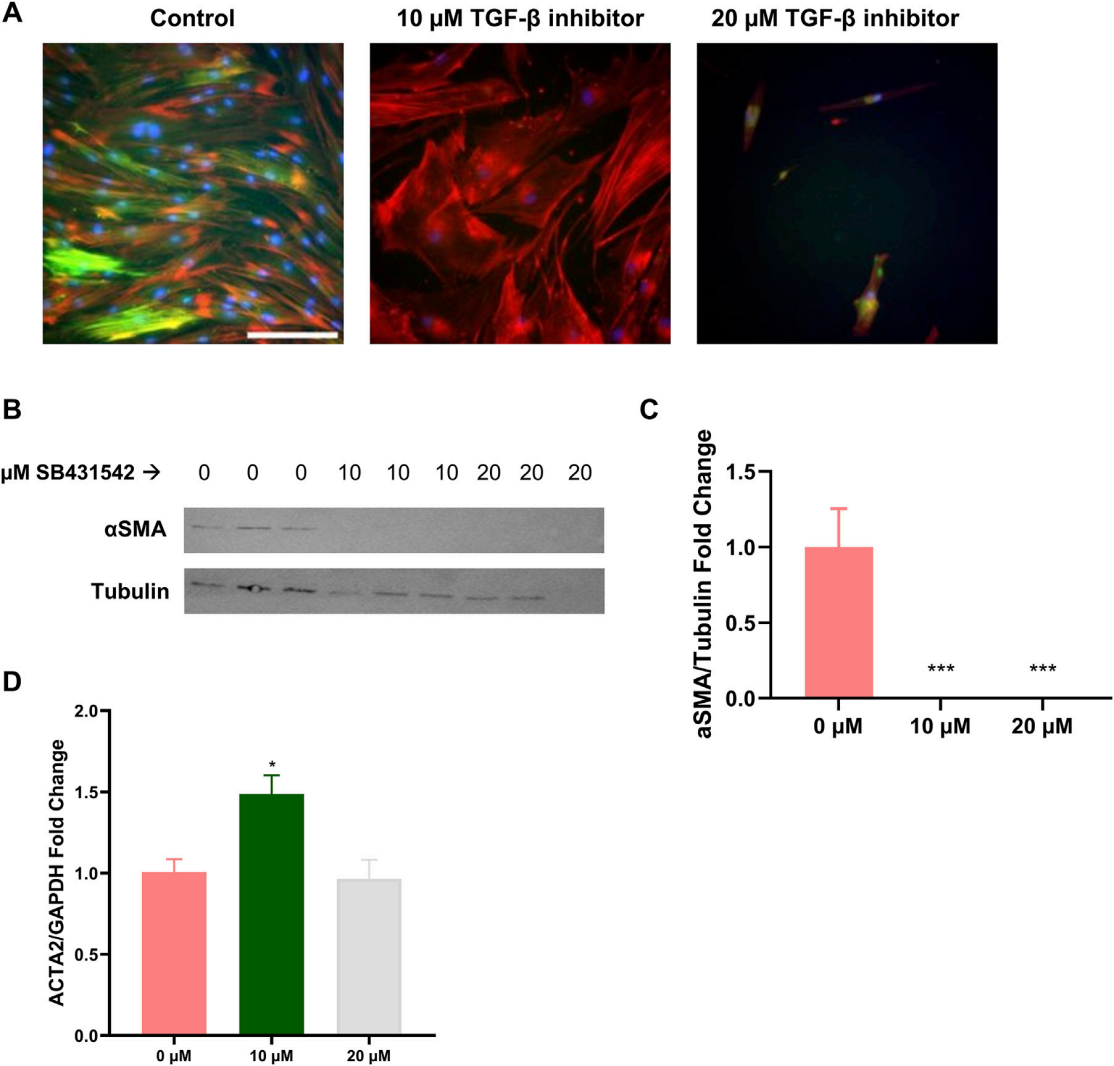
Effect of TGF-β inhibitor (SB431542) on cFB quiescence. **(A)** Immunofluorescence staining of α SMA (green), F-actin (red) and nuclei (blue) of cFbs at different TGF-β inhibitor concentrations. Scale bar: 100 μm. **(B)** Western blot images of α SMA protein expression and housekeeping protein (tubulin). **(C)** Quantification of the Western blot. **(D)** ACTA2 gene expression by qPCR-RT. n = 3 for all experiments. One-way ANOVA was used in **(C, D)**. * indicated significant differences compared to the 0 μM group.

**FIGURE 4:**
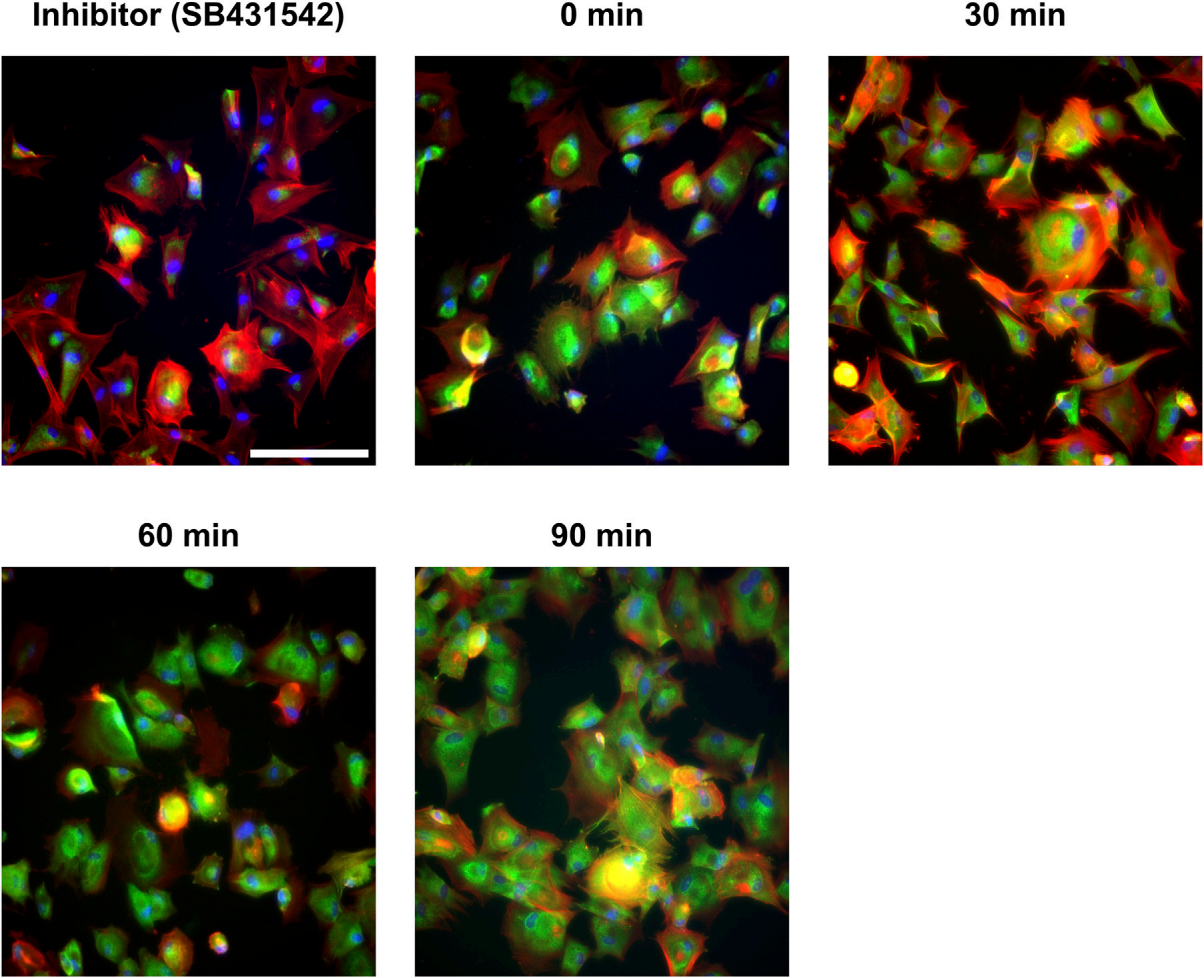
Immunofluorescence images of cFb activation post-TGF-β inhibitor removal at varied time points. α SMA (green), F-actin (red) and nuclei (blue). Scale bar = 100 μm. Representative images from n = 3 experiments.

The primary objective of GelMA hydrogels stiffening via a two-step photo-crosslinking process was to establish a dynamic mechanical environment for cell culture. This, to examine how variations in previously experienced substrate stiffness affected the activation status and ECM production of cFbs. However, cFbs were initially cultured and expanded on standard plastic culture plates, which have a very stiff substrate and potentially activate the cFbs to a more fibrotic state. Fibroblast activation is typically marked by the presence of αSMA stress fibers (Figure 3A). Directly using these activated cFbs in our GelMA hydrogel experiments could mask the effects of dynamic stiffness changes on cFb behavior. To address this, and before performing experiments on the GelMA hydrogels, we aimed to revert the cFbs to a more quiescent, mechanically naïve state. Our first approach involved investigating the impact of FBS concentration in cell culture medium on cFb activation. Consistent with prior findings (Siani et al., 2015), lower serum concentrations reduced αSMA expression in cFbs but significantly hampered cell proliferation (Supplementary Figure S1). As an alternative, we examined the use of a TGF-β inhibitor (SB431542), known for its ability to deactivate fibroblasts. Different concentrations of this inhibitor were tested, with 10 µM found to be most effective. This concentration successfully inhibited αSMA expression while preserving cell shape and proliferation (Figure 3A), in contrast to what has been observed with higher inhibitor doses (Hjelmeland et al., 2004; Koh et al., 2015). The decrease in αSMA was confirmed at the protein but not at gene expression levels (Figures 3B–D). This difference can be attributed to feedback mechanisms present in fetal-like cell types (Mahmood et al., 2010). Removing the inhibitor from the culture medium resulted in an immediate restoration of αSMA expression in the cFbs (Figure 4). In summary, by treating cFbs with the TGF-β inhibitor prior to seeding them on the GelMA hydrogels, we successfully prepared more quiescent and mechanically naïve cells for the experiments.

### cFbs activation and ECM related gene expression is affected by the previous stiffness

Quiescent-induced cFbs were initially seeded on top of GelMA hydrogels of varying stiffnesses (Soft, Med and Stiff static groups) without presence of TGF-β inhibitor. Cell viability was assessed, showing good viability at 24 h and 48 h, despite the presence of a considerable number of dead cells (Supplementary Figure S2). Furthermore, dynamically stiffened groups were further photo-crosslinked (at 24 h). This second photo-crosslinking did not produce any further cell damage (Supplementary Figure S2).

**FIGURE 5:**
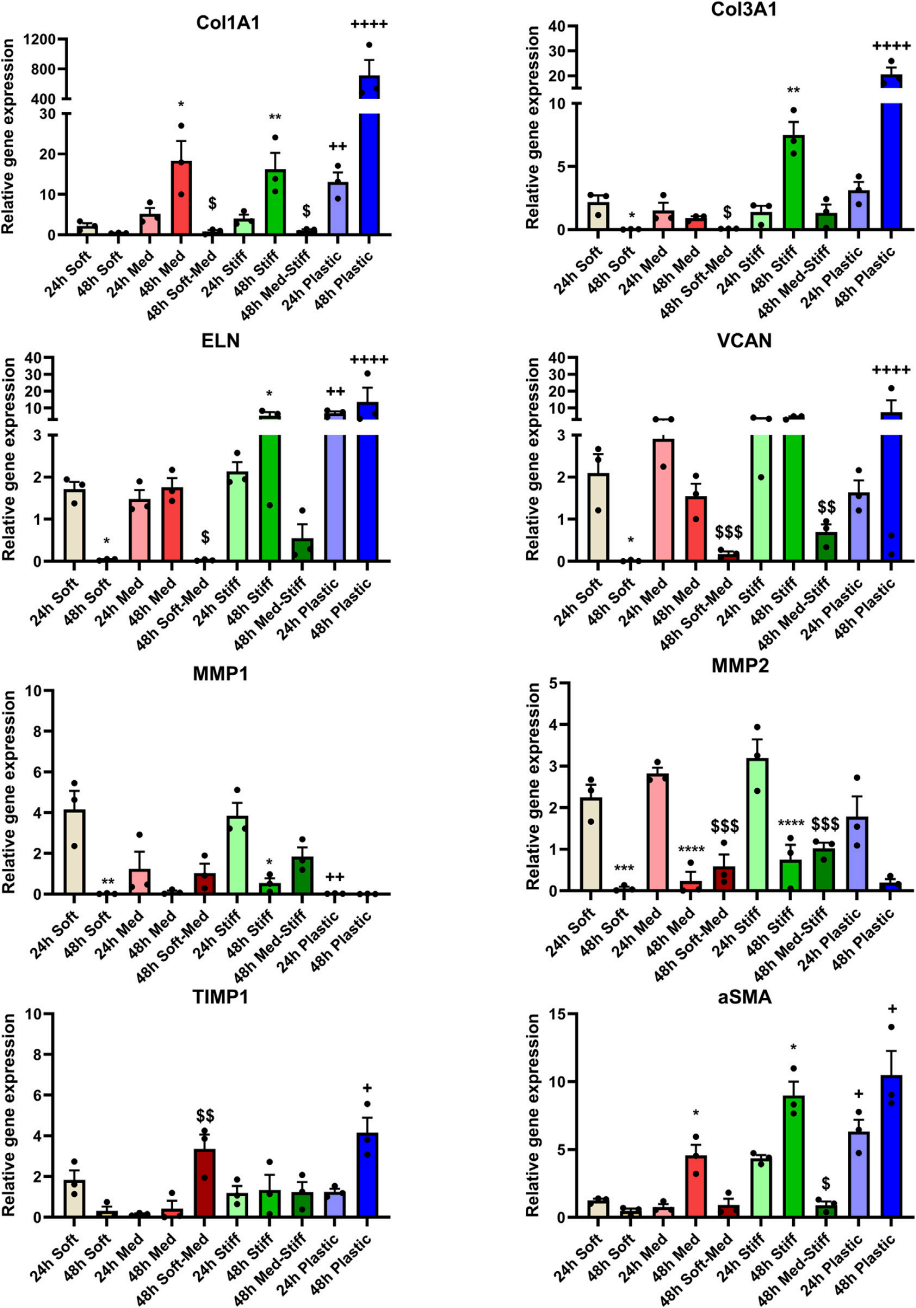
cFb activation and ECM gene expression is determined by the pas stiffness. Gene expression for markers related to cFb activation (αSMA), growth markers (i.e., collageneous matrix, proteoglycan, elastic matrix formation and basement membrane), as well as markers related to remodeling. *, **, ***, **** indicates significant differences between the 24 h and 48 h of the same stiffness group (Soft vs. Soft, Med vs. Med or Stiff vs. Stiff). $ , $$ , $$$ indicates significant differences between 48 h Soft-Med vs. 24 h Med or 48 h Med-Stiff vs. 24 h Stiff. +, ++, ++++ indicates significant differences between 24 h Plastic vs. 24 h Soft or 48 h Plastic vs. 48 h Soft.

After evaluating cFbs viaibility on GelMA hydrogels, we measured the gene expression related to cFbs activation and ECM markers at 24 h and 48 h. The genes studied were relative to growth markers (i.e., collagenous matrix, proteoglycans, elastic matrix, basement membrane), remodeling markers (i.e., MMPs and TIMPs) and cell activation (αSMA). Interestingly, control groups showed an increased gene expression at 48 h compared to 24 h for Col1A1, αSMA and decrease on MMP expression, directly related to a more cFbs activation and ECM producing phenotype for cFbs that were cultured on Med and Stiff (Figure 5) groups but the opposite trend was observed for the Soft group (Figure 5A). These results suggest that there is a stiffness threshold between ∼5 and ∼12 kPa that tends to activate cFbs to a more profibrotic state, whereas below this threshold, they tend to remain more quiescent. This hypothesis was further confirmed by measuring the gene expression of cFbs cultured on top of plastic substrate, that is typically orders of magnitude stiffer than *in vivo* ECM. Results showed that cFbs activation is much more pronounced, being the gene expression ∼20–50 times higher than Med and Stiff groups (Figure 5; Supplementary Figure S3). No major differences were observed in proteoglycan, elastic matrix and basement protein expression.

In contrast, cFbs seeded on GelMA hydrogels that underwent dynamic stiffening at 24 h (Figure 5) displayed a decreased gene expression at 48 h compared to the 24 h control group indicating that the initial 24 h at a lower stiffness mechanically preconditioned the cFbs to show a more quiescent phenotype. This effect was more pronounced in the Soft-Med group, as the Soft stiffness supported the quiescent state in cFbs. When the stiffness was changed to Med, the cFbs still partially retained this quiescent state. However, the changes were not as significant in the Med-Stiff group, since Med stiffness alone is already an activator of cFbs. In summary, environmental stiffness for Med and Stiff group is a potent cue to induce cFbs activation towards a more profibrotic state while the Soft stiffness supports cFb quiescence. This activation can be modulated by exposing cFbs to an initially lower stiffness before stiffening the environment.

## Discussion

*In vitro* models that dynamically tune the mechanical properties of cellular environments are crucial for deciphering cellular behavior mechanisms in pathological processes involving tissue remodeling, such as tumor progression or myocardial injury post-MI. GelMA hydrogels have been extensively studied for exposing cells in 2D and 3D mechanical environments with varying stiffnesses (Ding et al., 2019; Kong et al., 2020; Li et al., 2021). However, methods to create dynamic mechanical environments using GelMA remain unexplored. This study presents a method for dual photo-crosslinking GelMA hydrogels, thereby exposing cells grown on top of the hydrogels to dynamic mechanical environments. The system is used to investigate how the activation of cFbs activation phenotype is influenced by the previous substrate stiffness (applied for 24 h), providing insights into cFb mechanobiology in general and mechanical memory in particular.

Initially, the physical properties of GelMA hydrogel were characterized. In our experiments, GelMA concentrations of 10% and 15% resulted in significantly stiffer hydrogels (∼4-fold increase) compared to 5% concentration, reaching approximately 35 kPa (Figure 1B). The sol fraction, remaining between 15% and 30% across all concentrations and UV illumination durations (3–15 min), indicated unreacted methacrylate groups within the gelatin backbone. This characteristic suggested the potential for further photo-crosslinking these groups to increase hydrogel stiffness beyond its initial properties. While two-step photo-crosslinking of HAMA for 3D bioprinting applications has exploited the presence of unreacted methacrylate groups (Skardal et al., 2010), the potential of re-crosslinking these groups hours or days after the initial photo-crosslinking, especially in the presence of cell culture medium, remained unexplored.

In our study GelMA hydrogels that underwent a second photo-crosslinking step were supplemented with 0.1% LAP (photoinitiator) 45 min prior to illumination. This was crucial, as without additional LAP, further crosslinking did not occur (data not shown). This is because after keeping the hydrogels for 24 h in cell culture medium and 37°C, remaining LAP of the first illumination was depleted, necessitating the addition of extra photoinitiator for successful re-crosslinking. The second photo-crosslinking step effectively increased the initial stiffness of hydrogels from ∼8 kPa to ∼12 kPa (Soft-Med) and from ∼15 kPa to ∼40 kPa (Med-Stiff) (Figures 2B,C). Sol fraction results showed a reduction of unreacted methacrylate groups from 20%–30% to 1%–5%. Similar to this approach, a recent study used the unreacted methacrylate groups in HAMA hydrogel to create a dynamic stiffness environment to study malignancy in tumor cells (Ondeck et al., 2019).

Techniques to dynamically change the extracellular environment usually consist of ECM molecules modified with stimuli-responsive motifs. The stimuli, such as temperature (Kim et al., 2010), light (Rosales et al., 2017), magnetic force (Zhou et al., 2023), and biomolecules (Straley and Heilshorn, 2009), have been employed to trigger dynamic changes in stiffness. These methods generally offer more versatility in tuning cell environmental stiffness but require extensive chemical knowledge and advanced techniques. In contrast, the method presented here is straightforward and easily implementable in laboratories with less expertise in chemistry, yet still wishing to address relevant questions in mechanobiology and cellular mechanical memory.

A notable aspect of our study was the focus on inducing a quiescent state in cFbs prior to their exposure to GelMA hydrogels of varying stiffness. This step ensured that the observed cellular responses were attributable to the mechanical environment, not residual effects from previous culture conditions. Fibroblasts are known to be highly sensitive to substrate stiffness, with stiffer substrates typically inducing a more activated, fibrotic state (Pesce et al., 2023). Since cFbs in our study were expanded in standard plastic flasks (very stiff substrates), a mechanical memory could have been introduced by the flask substrate, potentially biasing our results. Initially, we explored reducing FBS concentration in the cell culture media from 10% to two or 0%. The effect of reducing FBS achieved to reduce cellular activation through the decrease of αSMA stress fibers, in agreement with other studies (Supplementary Figure S1; (Baranyi et al., 2019; Zhang et al., 2019; Pesce et al., 2023; Ramklowan et al., 2023)). However, as reported by other studies, this reduction compromises cellular viability (Rashid and Coombs, 2019; van Vijven et al., 2021). Alternatively, using a TGF-β inhibitor proved effective in achieving a quiescent state in cFbs, aiming to induce a more mechanically naïve cell type, thereby enhancing the relevance of our findings regarding mechanical memory. A 10 µM concentration of TGF-β inhibitor completely inhibited αSMA fiber presence while maintaining cFbs viability and morphology (Figure 3) (Zhang et al., 2019). As cFb were treated with the inhibitor while plated on stiff plastic culture plates, the removal of the inhibitor produced an immediate αSMA production and cFb activation (Figure 4). The effect of the inhibitor preventing aSMA fiber formation has been described as a marker of low ECM-producing fibroblasts and mechanically naïve cells, thereby strengthening the hypothesis of cFbs being free of mechanical memory before seeding on the GelMA hydrogels (Kirk et al., 2021).

Our results highlight the significant role of mechanical history in determining cFb activation and ECM-related gene expression. Cells cultured on softer substrates (∼8 kPa, Soft group) remained more quiescent, whereas those on stiffer (∼35 kPa, Stiff group) substrates showed increased activation (Figure 5) (Blokland et al., 2022; Felisbino et al., 2024). cFbs cultured on intermediate stiffness (∼12 kPa, Med group) also showed an activated phenotype compared to the Soft group, suggesting a potential stiffness threshold for cFb activation. Several studies have shown that stiffness-mediated fibroblast activation is regulated by YAP translocation into the nucleus (Niu et al., 2020), with a suggested threshold in the range of ∼10–15 kPa (Scott et al., 2021). Additionally, our findings suggest that cells retain a ‘mechanical memory’ of their substrate’s stiffness. Notably, cFbs cultured for 24 h on soft hydrogel (Soft) remained more quiescent when the hydrogel was stiffened (Med). This aligns with studies in mechanical memory of fibroblasts which demonstrated that fibroblasts seeded in stiff substrates remained more activated after transferring them onto soft substrates compared to the non-preconditioned fibroblasts (Balestrini et al., 2012). Similar to our study, previous research has shown that fibroblasts initially cultured on soft substrates showed less activation when transferred to stiffer ones (Fan et al., 2017; Dunham et al., 2020).

Our study provides initial insights into the impact of substrate stiffness history on ECM related gene expression of cFb. Our approach paves the way for further research into the time and dose-dependent effects of stiffness history on cFb behavior. A significant avenue for future investigation involves understanding the duration cFbs maintain the effects of prior mechanical environments, particularly concerning cellular activation and ECM production. Additionally, examining the distinct impacts of varying the duration and intensity of stiffness preconditioning on cFb responses is crucial. It remains to be determined if altering the exposure length to a specific stiffness level or modifying the stiffness degree could enhance or extend the effects observed in cFb behavior (Yang et al., 2014).

In this study, we acknowledge several technical limitations that warrant further investigation. The small sample size (N = 3) in our ECM gene expression experiments was intended as an initial demonstration of our method’s potential. To build upon these preliminary insights, larger-scale studies are necessary to validate and expand our findings. Moreover, the extraction of RNA from gel-cell constructs proved challenging, highlighting a need for optimized protocols that may include adjustments in gel size and cell density. Finally, the thickness of our constructs impeded effective fluorescent imaging, suggesting that modifications in sample preparation or imaging techniques may be required. Addressing these limitations will refine the current methodology providing a more robust platform for future research.

In summary, we propose a straightforward and effective method for dynamically modulating the mechanical properties of GelMA hydrogels, easily accessible to any cellular laboratory equipped with a UV light source. Our results emphasize the significance of mechanical memory in the activation of cardiac fibroblasts and ECM production, potentially advancing our understanding of cardiac tissue remodeling and pathology.

## Data Availability

The original contributions presented in the study are included in the article/Supplementary Material, further inquiries can be directed to the corresponding authors.
