# Supplementary material for: GelMA hydrogel dual photo-crosslinking to dynamically modulate ECM stiffness
Source: Front Bioeng Biotechnol. 2024 Jun 20;12:1363525. doi: 10.3389/fbioe.2024.1363525 (PMC11222782; doi:10.3389/fbioe.2024.1363525)
Supplement: Supplementary file 1 [file Table1.DOCX]

**Table 1.** Summary of the performed experiments for GelMA hydrogels.

|  | **GelMA concentration** | **First illumination time point** | **UV illumination time** | **Second illumination time point** | **UV illumination time** | **Performed experiments** |
| --- | --- | --- | --- | --- | --- | --- |
| **Static stiffness GelMA hydrogel characterization** | 5% | 0h | 5s – 20 min | --- | --- | Stiffness, Swelling, Sol fraction |
|  | 10% | 0h | 5s – 20 min | --- | --- | Stiffness, Swelling, Sol fraction, Cell culture, SEM |
|  | 15% | 0h | 5s – 20 min | --- | --- | Stiffness, Swelling, Sol fraction |
| **Dynamic stiffness GelMA hydrogel characterization** | 10% | 0h | 5s | 24h (dynamic stiffening) | 5 min | Stiffness, Swelling, Sol fraction, Cell culture, SEM |
|  | 10% | 0h | 15s | 24h (dynamic stiffening) | 5 min | Stiffness, Swelling, Sol fraction, Cell culture, SEM |
