# Supplementary figures and images for: GelMA hydrogel dual photo-crosslinking to dynamically modulate ECM stiffness
Source: Front Bioeng Biotechnol. 2024 Jun 20;12:1363525. doi: 10.3389/fbioe.2024.1363525 (PMC11222782; doi:10.3389/fbioe.2024.1363525)

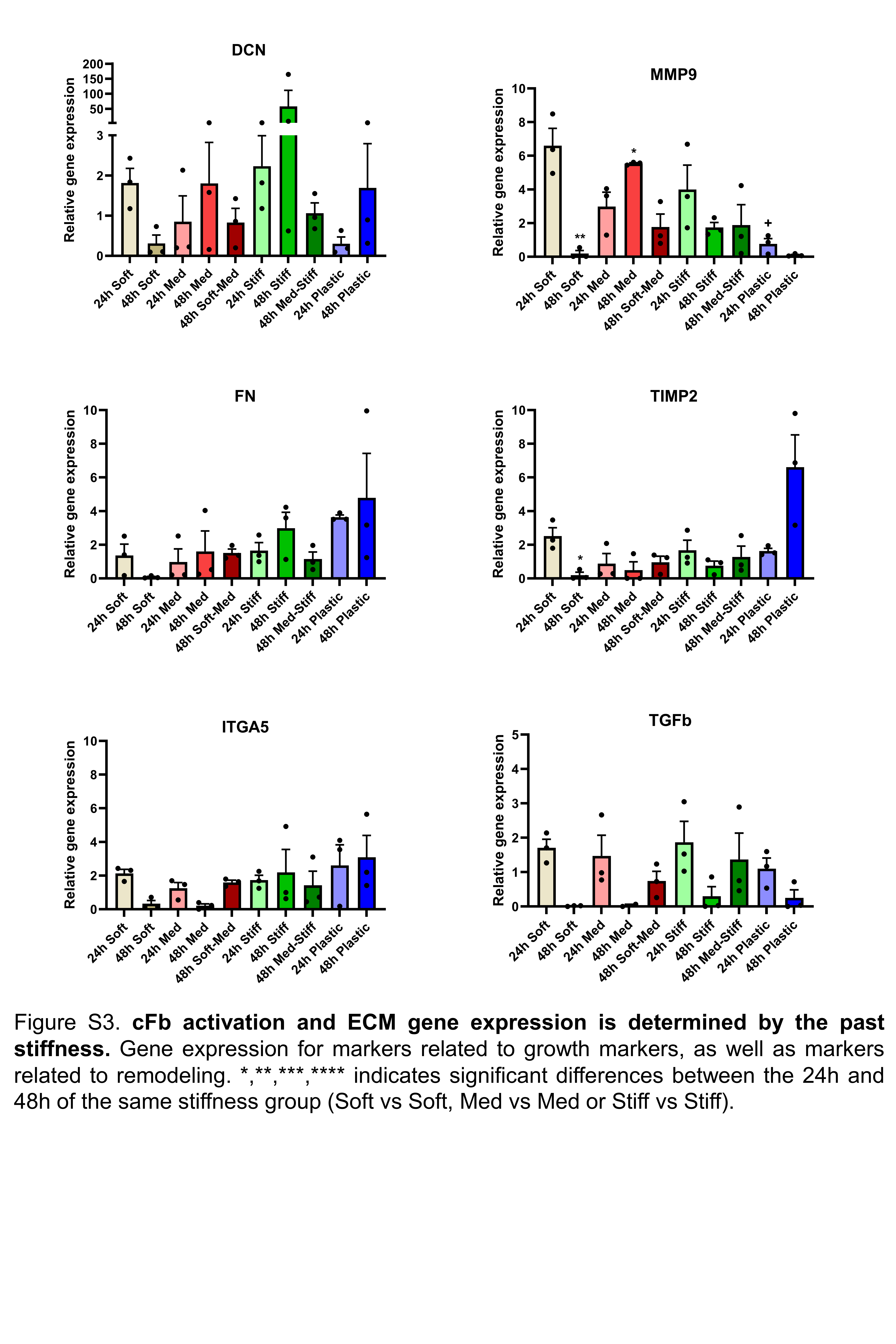

Supplement: Supplementary file 2 [file Image3.tif]

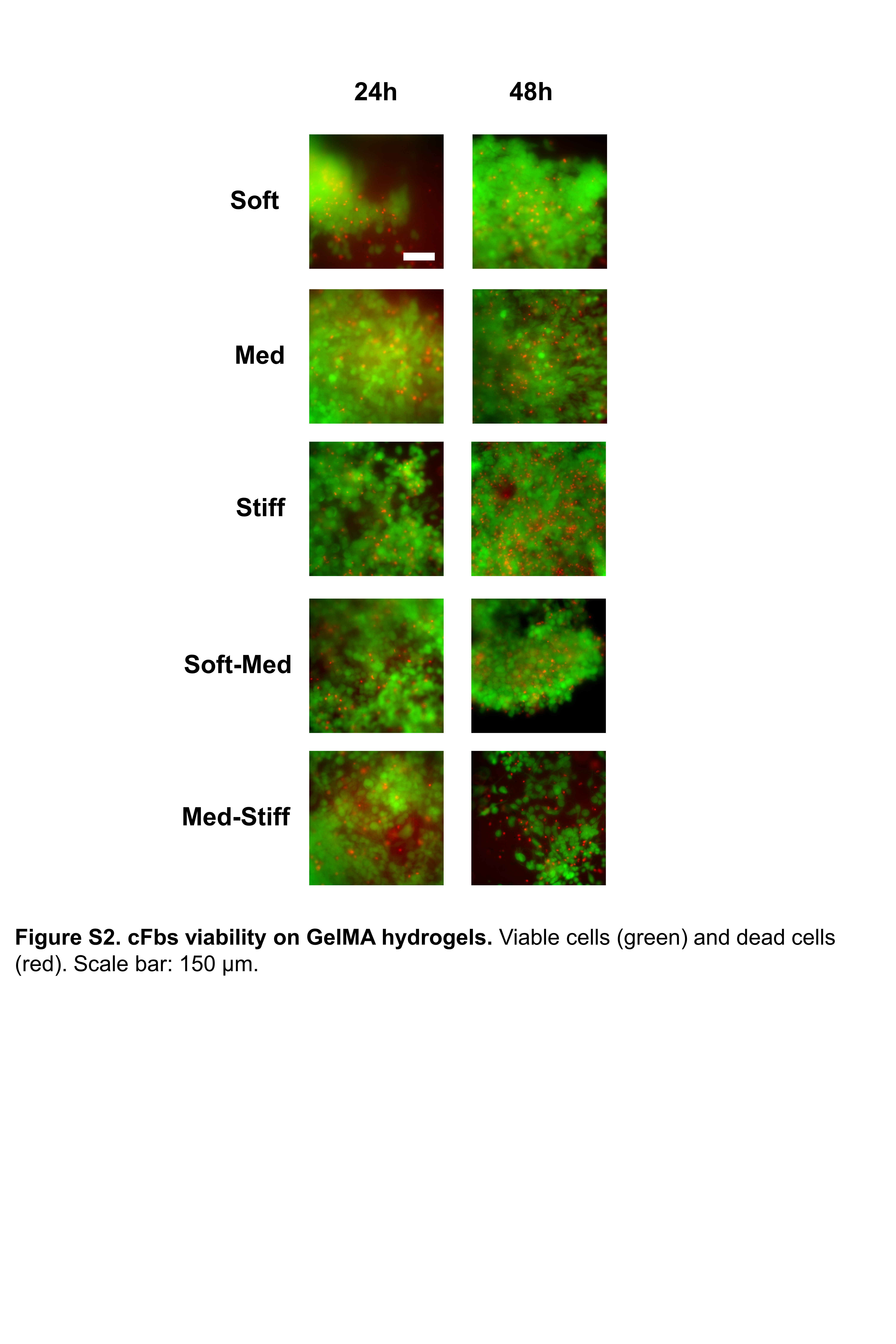

Supplement: Supplementary file 3 [file Image2.tif]

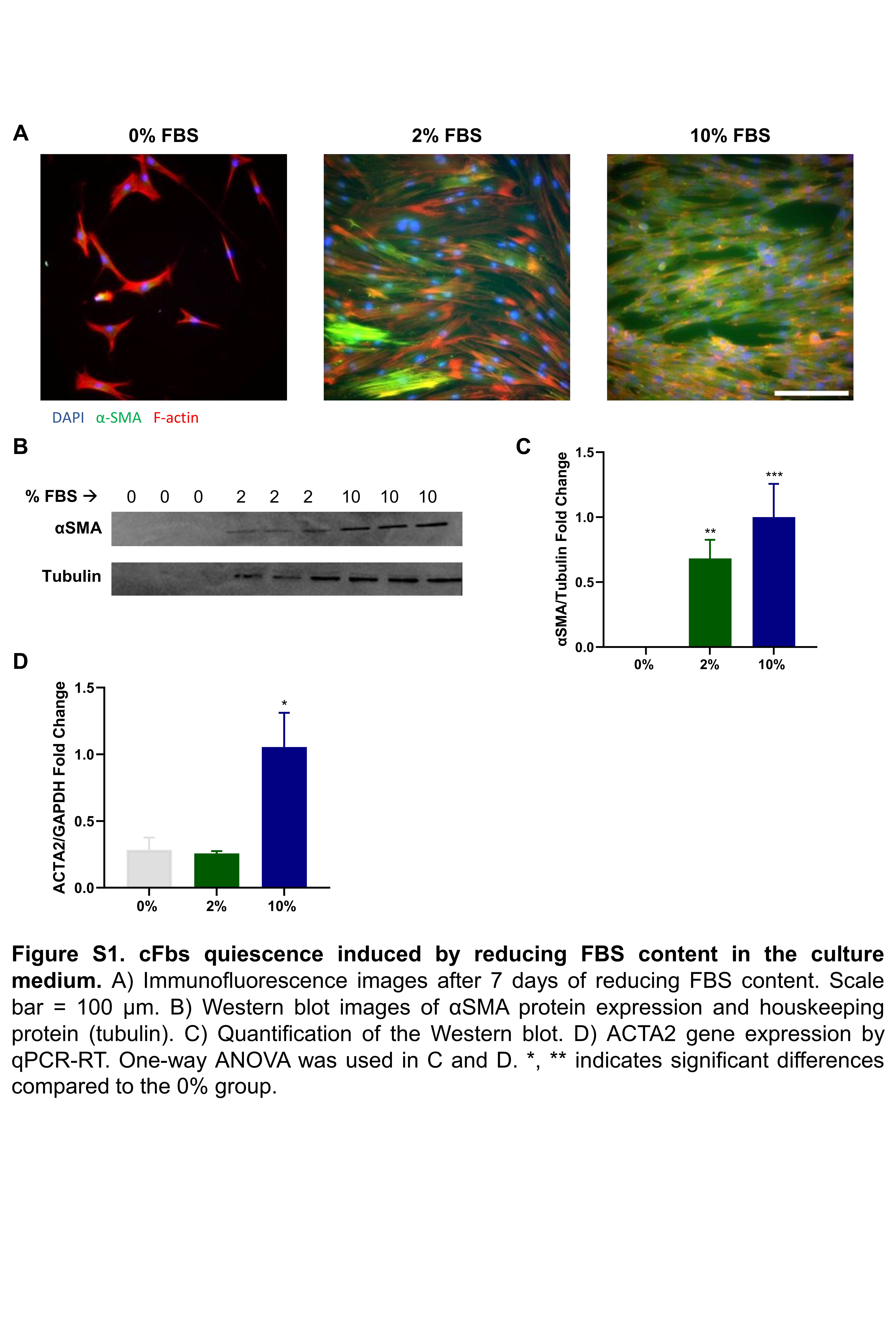

Supplement: Supplementary file 4 [file Image1.tif]
