# Supplementary material for: GelMA hydrogel dual photo-crosslinking to dynamically modulate ECM stiffness
Source: Front Bioeng Biotechnol. 2024 Jun 20;12:1363525. doi: 10.3389/fbioe.2024.1363525 (PMC11222782; doi:10.3389/fbioe.2024.1363525)
Supplement: Supplementary file 5 [file Table2.DOCX]

**Table S1.** RT-qPCR primers

| **Gene** | **Primer Set** | **Code 5’ – 3’** | **Application size (bp)** | **Position** | **Efficiency** |
| --- | --- | --- | --- | --- | --- |
| ACTA2 | FW  RV | CGTGTTGCCCCTGAAGAGCAT  ACCGCCTGGATAGCCACATACA | 134 | 336-469 | 105% |
| Col 1A1 | FW  RV | AATCACCTGCGTACAGAACGG  TCGTCACAGATCACGTCATCG | 86 | 3049-3134 | 100.5% |
| Col 3A1 | FW  RV | ATCTTGGTCAGTCCTATGC  GGAATTTCTGGGTTGGG | 140 | 218-357 | 106% |
| DCN | FW  RV | AATGCCATCTTCGAGTGGTC  TGCAGGTCTAGCAGAGTTGTGT | 91 | 466-556 | 102% |
| ELN | FW  RV | CTGGAATTGGAGGCATCG  CCTGGGACACCAACTAC | 146 | 1268-1413 | 99% |
| FN | FW  RV | AAGACCAGCAGAGGCATAAGG  CACTCATCTCCAACGGCATAATG | 136 | 6975-7110 | 100% |
| GAPDH | FW  RV | GGTGAAGGTCGGAGTCAACG  GCTTCCCGTTCTCAGCCTT | 191 | 85-275 | 100.2% |
| ITGA5 | FW  RV | TGCCTCCCTCACCATCTTC  TGCTTCTGCCAGTCCAGC | 171 | 1556-1726 | 104.7% |
| MMP 1 | FW  RV | CGCACAAATCCCTTCTACCC  CTGTCGGCAAATTCGTAAGC | 101 | 901-1001 | 105% |
| MMP 2 | FW  RV | ATGACAGCTGCACCACTGAG  ATTTGTTGCCCAGGAAAGTG | 174 | 1074-1247 | 98% |
| MMP 9 | FW  RV | TGGGGGGCAACTCGGC  GGAATGATCTAAGCCCAG | 224 | 1032-1255 | 101% |
| TGF-β | FW  RV | CGAGCCCTGGACACCAACT  TGGCATGGTAGCCCTTGGG | 127 | 1671-1797 | 100% |
| TIMP 1 | FW  RV | TGACATCCGGTTCGTCTACA  TGCAGTTTTCCAGCAATGAG | 102 | 429-530 | 92% |
| TIMP 2 | FW  RV | GGAGGAATCGGTGAGGTC  AACAGGCAAGAAGCAATGG | 104 | 1734-1837 | 97% |
| VCAN | FW  RV | GGCACCTGTTATCCTACTGAAA  ACACAAGTGGCTCCATTACG | 125 | 9660-9784 | 96% |
